# Interactions between Muscle and Bone—Where Physics Meets Biology

**DOI:** 10.3390/biom10030432

**Published:** 2020-03-10

**Authors:** Marietta Herrmann, Klaus Engelke, Regina Ebert, Sigrid Müller-Deubert, Maximilian Rudert, Fani Ziouti, Franziska Jundt, Dieter Felsenberg, Franz Jakob

**Affiliations:** 1 Orthopedic Department, Bernhard-Heine-Center for Locomotion Research, IZKF Research Group Tissue regeneration in musculoskeletal diseases, University Hospital Würzburg, University of Wuerzburg, 97070 Würzburg, Germany; m-herrmann.klh@uni-wuerzburg.de; 2 Department of Medicine 3, FAU University Erlangen-Nürnberg and Universitätsklinikum Erlangen, 91054 Erlangen, Germany; klaus.engelke@imp.uni-erlangen.de; 3 Orthopedic Department, Bernhard-Heine-Center for Locomotion Research, University of Würzburg, IGZ, 97076 Würzburg, Germany; r-ebert.klh@uni-wuerzburg.de (R.E.);; 4 Orthopedic Department, Bernhard-Heine-Center for Locomotion Research, University of Würzburg, 97074 Würzburg, Germany; m-rudert.klh@uni-wuerzburg.de; 5 Department of Internal Medicine II, University Hospital Würzburg, 97080 Würzburg, Germany; Ziouti_F@ukw.de (F.Z.); Jundt_F@ukw.de (F.J.); 6 Privatpraxis für Muskel- und Knochenkrankheiten, 12163 Berlin Germany; dieter.felsenberg@charite.de

**Keywords:** muscle, bone, mechanosensing, mechanotransduction, myokines, osteokines adaptation

## Abstract

Muscle and bone interact via physical forces and secreted osteokines and myokines. Physical forces are generated through gravity, locomotion, exercise, and external devices. Cells sense mechanical strain via adhesion molecules and translate it into biochemical responses, modulating the basic mechanisms of cellular biology such as lineage commitment, tissue formation, and maturation. This may result in the initiation of bone formation, muscle hypertrophy, and the enhanced production of extracellular matrix constituents, adhesion molecules, and cytoskeletal elements. Bone and muscle mass, resistance to strain, and the stiffness of matrix, cells, and tissues are enhanced, influencing fracture resistance and muscle power. This propagates a dynamic and continuous reciprocity of physicochemical interaction. Secreted growth and differentiation factors are important effectors of mutual interaction. The acute effects of exercise induce the secretion of exosomes with cargo molecules that are capable of mediating the endocrine effects between muscle, bone, and the organism. Long-term changes induce adaptations of the respective tissue secretome that maintain adequate homeostatic conditions. Lessons from unloading, microgravity, and disuse teach us that gratuitous tissue is removed or reorganized while immobility and inflammation trigger muscle and bone marrow fatty infiltration and propagate degenerative diseases such as sarcopenia and osteoporosis. Ongoing research will certainly find new therapeutic targets for prevention and treatment.

## 1. Introduction

**Figure 1:**
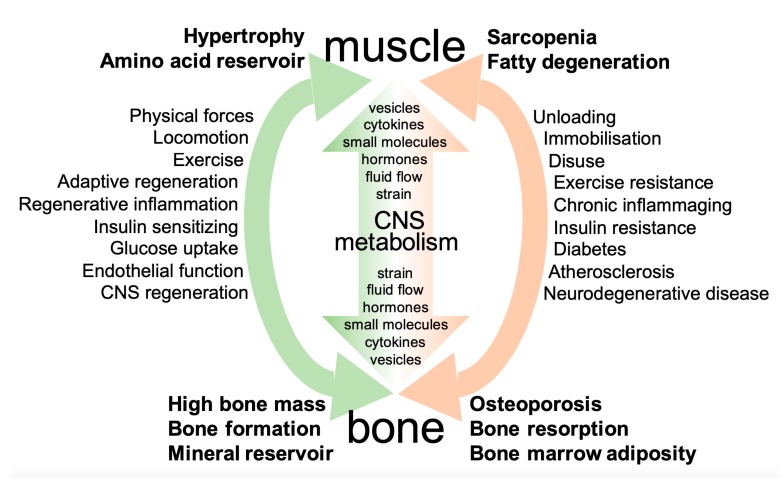
Schematic overview of the interaction between muscle and bone in physiology and pathology, addressing also the most important effectors of interaction. The green color represents the physiological interactions indicating that muscle hypertrophy and high bone mass have interdependent relationships, while the red color shows similarly strong mutual interaction between the loss of bone and muscle due to the intrinsic pathology in degenerative disease such as osteoporosis and sarcopenia, but also lifestyle, disuse, and underlying metabolic diseases such as chronic inflammatory conditions and diabetes mellitus.

Bone and muscle are both tissues of mesodermal origin that together with joints constitute the musculoskeletal functional unit to facilitate the locomotion of the organism, which is guided by the central nervous system and the neuronal networks and their neuromuscular junctions. Both tissues provide a highly flexible system of adaptation as a response to the variability of physical forces generated through gravity and environmental conditions. This influences tissue structures from development throughout adult life using similar principles of both tissue formation and repair/regeneration. Evolution has provided multiple pathways for the mutual interaction of muscle and bone in order to adapt the stability of the skeleton and muscle to the forces needed for locomotion and environmental challenges. Recent information indicates that the reciprocity of mechanochemical interaction is extremely dynamic, including the cellular, the tissue, and the organismic level [1,2,3,4]. Intrinsic as well as extrinsic forces such as gravity generate adaptive changes of tissues, which modulate muscle power and fracture resistance. Many interactions are not only bilateral but follow a multilateral or even organismic relationship, involving metabolism and the nervous system (Figure 1).

In pathology and in aging, accumulating cell and tissue damage can alter the delicate balance of adaptive maintenance and regeneration and may cause disease and degenerative conditions. This review summarizes knowledge about the principles of mutual interactions of muscle and bone, where physics and biochemistry/cell biology get integrated and adjust to each other according to environmental needs. It also highlights the common effectors of these interactions and the secondary response, which sustains a dynamic reciprocity of adaptation. Connective tissues physically joining muscle and bone, especially tendons and ligaments, naturally play a critical role in this interaction, as addressed in a recent review article in detail [5].

## 2. Principles of Interaction between Muscle and Bone

### 2.1. Physical Forces

#### 2.1.1. Forces Generated by Exercise, Locomotion, and External Vibration

Due to gravity on earth, organisms must adapt their constitution to extrinsic physical forces from the beginning. Mobile forms of life in addition generate intrinsic forces when moving. Human beings developed the capacity of locomotion during evolution and adapted to the daily needs for hunting and gathering, which required climbing trees and hills but also running on plain territory. In modern societies, exercise and sports have largely replaced the ancient motivations of locomotion. The physical forces generated in the context of motion are extremely variable from very low to high impact. The variability of impact on the different levels of cell/cell, cell/tissue, and tissue/tissue interaction ranges from 0.1 to 100 kPa from development to adult life and produces a complex interactive network of physics and biochemistry involving both muscle and bone [3,6,7,8].

**Figure 2:**
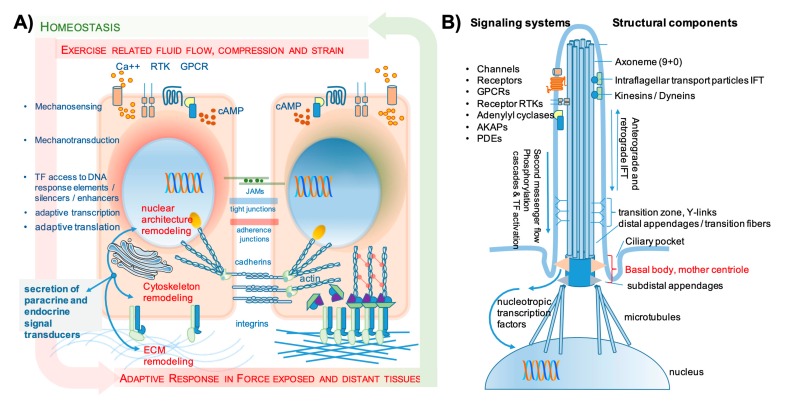

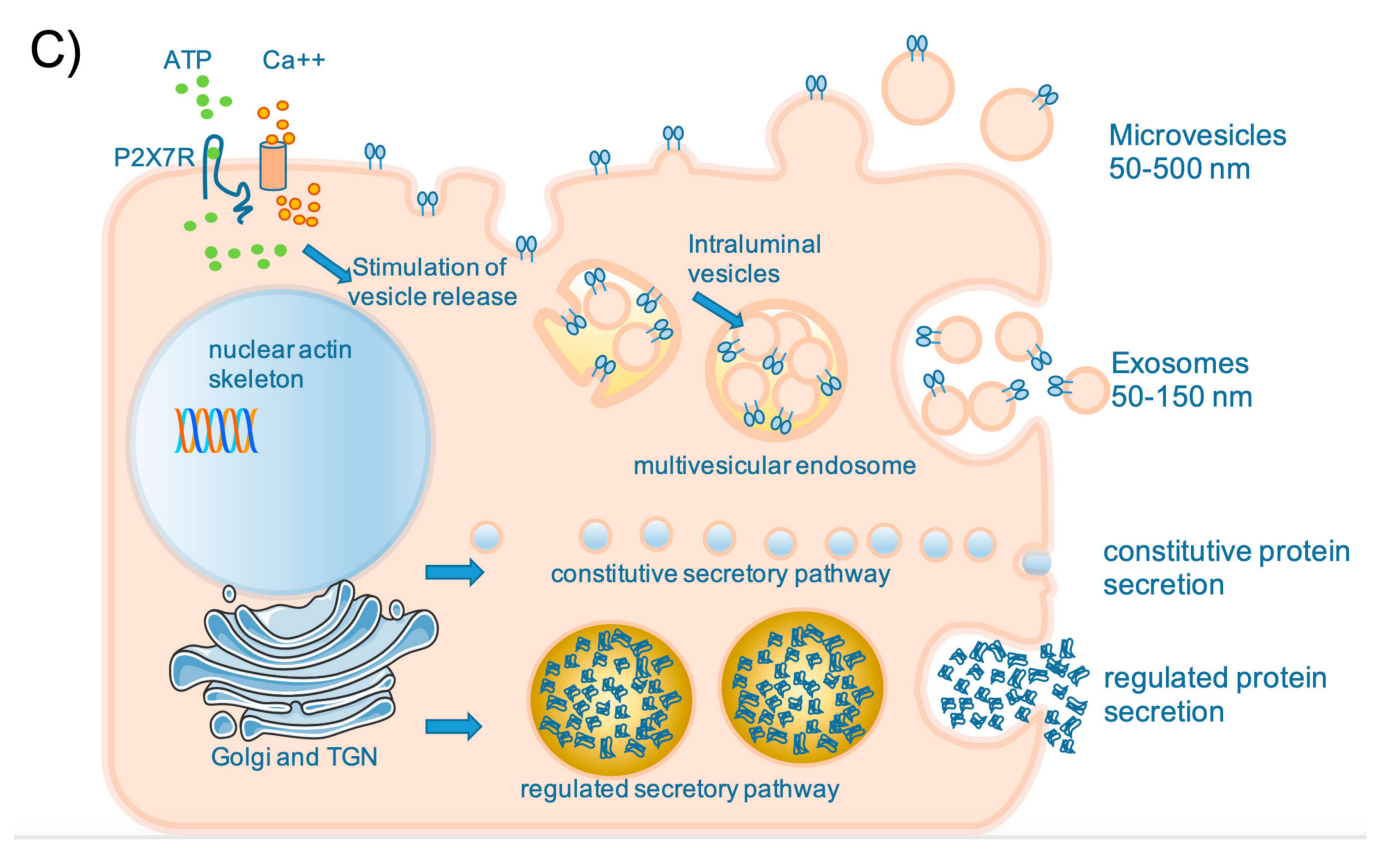
Mechanisms of mechanotransduction. Graphical summary of the current knowledge on the transduction of mechanical signals into a cellular response. **A**) Mechanical strain applied to the cell and (the resulting) fluid flow activate receptors and channels and their downstream signaling cascades. In a tissue context, strain is transmitted between cells via a series of adhesion molecules such as integrins, cadherins, and tight junctions. Transcription factors become nucleotropic and address DNA regulatory elements such as repressors, enhancers, and their specific DNA response elements. Adaptive transcription and translation are initiated and lead to changes in transcriptome, proteome, and especially the secretome to communicate with neighbors and distant tissues. The expression and secretion of extracellular matrix proteins as parts of the secretome are upregulated. As a consequence, the incoming forces and the resistive response to external forces are enhanced, and a new homeostasis situation is produced. Similarly, the production of proteins of the cytoskeleton is enhanced, the cytoskeleton is also becoming stiffer, and the contractile proteins generate even more active forces. **B**) Fluid flow is sensed by the primary cilium. The relevant structural features of a primary cilium are schematically depicted on the right side, while the signaling cascades and tools for mechanotransduction are depicted on the left side. Deflection of the cilium generates signaling [43,44]. Intraflagellar transport is a means of active transport of signaling peptides to and from the cell body. **C**) The constitutive secretory pathway and the regulated secretory pathway are schematically depicted. While the latter has not been demonstrated in muscle or bone, it is characteristic for truly endocrine cells and requires complex sorting and the intravesicular storage of preformed proteins that are extruded upon endocrine signals. The constitutive pathway requires characteristic secretory peptide sequences to be continuously released into the extracellular environment and subsequently the circulation. The release of vesicles upon exercise-related calcium flux into the cell has been demonstrated to be a candidate mechanism for the release of proteins and miRNAs. Abbreviations: RTK: receptor tyrosine kinase; GPCR: G-protein coupled receptor; ATP: adenosine triphosphate; cAMP: cyclic adenosine-monophosphate; ECM: extracellular matrix; AKAPs: A-kinase anchoring proteins; PDEs: phosphodiesterases; P2X7R: purinergic receptor P2X, ligand-gated ion channel; TGN: trans-Golgi-network; This figures was created using inspiring information and cartoons from [6,9,40,44,45,46,47,48,49,50,51].

Mechanical stimuli can be transmitted through deformation (stretching and compression) and/or shear strain applied by fluids that pass by. The former prevail in muscle while bone strongly (but not exclusively) responds to fluid flow in the osteocyte canalicular network [9]. Mechanochemical coupling mechanisms translate the respective adaptation of living structures to physical challenges. In response to forces, all cells and tissues adapt their resistive forces as mediated through the strength and stiffness of their cytoskeleton and extracellular matrix (ECM). The clinical result of physical impact is high bone and muscle mass, resulting in enhanced fracture resistance and muscle power (see also Figure 2A,B). Moreover, the weight-saving mode of construction of bone architecture allows for local adaptations to the directions of incoming forces (force lines); these are phenomena that for bone have been described earlier as Wolff’s law, in which form follows function [10,11].

The physical mechanisms of applying mechanical forces on living organisms such as gravity and active muscle contraction have recently been complemented by technically applied forces generated by external vibrations of different amplitudes and frequencies. Two different modes of operation exist: one type of vibration plates uses the alternating vibration, which provokes reactive muscle contractions up to around 30 Hz with variable amplitudes. The other device type applies low-amplitude/high-frequency vibration without alternation. Both types of machines have been introduced commercially and partly ahead of scientific evaluation. However, the evidence-based knowledge has been increased during the last decade. There is no doubt that external mechanical vibration may variably substitute for gravity and add up to active muscle contraction both in space missions and as dedicated types of exercise. Vibration certainly can also elicit cellular responses, which may be beneficial in terms of inducing and maintaining muscle hypertrophy and elevated bone mass under conditions of disuse or unloading in simulated microgravity [12,13,14]. Nevertheless, much more research is needed to further explore the beneficial versus harmful effects in health and disease. Above all, this applies to conditions where exercise- and mechanoresistance or the dysregulation of mechanoresponse are part of the disease or of aging-related changes [12,15,16,17]. For the time being, it is probably correct to state that vibration techniques if correctly applied may help to delay disuse-related and microgravity-related muscle and bone loss, but the specific molecular mechanisms of exercise resistance in disease have still to be unraveled to efficiently fight the respective aging-associated diseases.

#### 2.1.2. Mechanosensing and Mechanotransduction

At the cellular level, the reciprocity of continuous challenge and adaptation has provoked the statement that “virtually every step of cell and tissue biology depends upon mechanochemical events” as made by researchers who mainly deal with principles of cell migration [18]. The last three decades have seen an emancipation of mechanobiology as a modulator of cellular biochemistry, which is highly relevant for tissue formation already early in development and throughout the adult life cycle for tissue maintenance, remodeling, and regeneration [1,19]. Mechanical forces and cellular deformations can now be measured down to the molecular level, and their biological consequences are being unraveled, demonstrating a new world of interaction between physics and cell biology [4,20,21].

Cellular deformations induce adaptive signaling that provides adequate function, fulfilling the actual environmental needs/challenges. [22,23,24,25,26]. The opening of membrane channels followed by calcium influx or activation of enzymes such as adenylate or guanylate cyclase and the production of cyclic adenosine or guanosine monophosphate (cAMP or cGMP) are part of such intracellular signaling cascades. Integrin-mediated mechanotransduction is but one of many molecular mechanisms that appear to be linked to successful muscle regeneration in health, aging, and disease [27]. Connexins and pannexins are other adhesion mediators that form gap junctions and are involved in mechanotransduction and consecutive ATP secretion in both bone and muscle, especially as an important system in osteocytes. Connexin 43 has functions beyond the physical mechanotransduction between cells, since its knockout in early osteoblasts causes impaired muscle formation in mice [28,29].

Mechanotransduction even involves the nuclear envelope and nuclear pore structures as an upcoming new field of mechanobiological coupling, where deformations are followed by changes in the passage of molecules in and out of the nucleus and also changes in chromatin packing and architecture [30,31]. A recent review describes the microenvironmental changes in both osteocytes and muscle stem cells in response to mechanical cues, with a special focus on the respective cellular microenvironment. Sequential stages of response are described such as the secretion of nitric oxide (NO) and prostaglandins, the formation of integrin clusters with connection to intracellular anchoring complexes, the rearrangement of actin and tubulin to maintain cell morphology, and finally changes in nucleus morphology and volume (Figure 2A) [32].

Many molecular mechanisms of mechanosensing and mechanotransduction are common pathways for muscle and bone. Focal adhesion kinases, integrin-related adhesion, or Hippo/Yap/Taz signaling (Yes-associated protein (YAP); transcriptional coactivator with PDZ-binding motif (TAZ)) are just a few examples that recently have been characterized [32,33]. Specific mechanisms for muscle may involve titin, while calcium channels are fundamental in bone mechanotransduction (as reviewed in [34]). For muscle cells, new aspects become relevant after a recent report about caveolin 3 (Cav3) mutations, which cause various forms of myopathies. In human myoblasts, Cav3 deficiency was associated with the hyperactivation of IL-6-STAT3 signaling (interleukin-6, signal transducer and activator of transcription 3), which is a pathway that in the presence of WT Cav3 turned out to be regulated by mechanical strain in a Cav3-dependent manner [35]. Further research is necessary to describe the functional consequences for the respective cells and for tissue adaptation and regeneration, especially those that drive muscle hypertrophy upon mechanical loading.

Fluid flow is also sensed via adhesion molecules and adhesion complexes (transmembrane integrins, cadherins, connexins), the cytoskeleton, lipid rafts, ion channels, and in addition by the primary cilium. With slight variability concerning differential channel expression, virtually all bone cells express the necessary tools, including the primary cilium. The evidence for the relevance of fluid flow and deflection of primary cilia comes from in vitro studies but also from knockout (KO) mouse experiments that impair primary cilia development, but there is still some debate about the contributions and their molecular mechanisms related to primary cilia in mechanotransduction elicited by fluid flow [9,36,37,38,39]. Within the huge network of cellular dendritic processes of osteocytes, there is regular fluid flow, which also supports nutrient and waste transport. In addition, osteocytes regularly undergo deformations and retract and extend their processes, thus receiving mechanical triggers for activating mechanosensitive calcium channels. Depending on the amplitude and frequency of mechanical strain, they initiate and orchestrate regeneration and remodeling if needed. Bone cells are reported to express stress-activated ion channels (SAC, including the DEG/ENAC family of cation channels (named after the *Caenorhabditis elegans* degenerins and the mammalian epithelial Na^+^ channels), transient receptor potential (TRP) channels, L-type (osteoblasts) and T-type (osteocytes) voltage sensitive calcium channels (VSCC), Annexin V voltage-gated calcium channels (VGCC), and Piezo-type mechanosensitive ion channel component (Piezo 1 and 2). Mechanical stimulation enhances calcium flux into the cell (reviewed in [22]), and the resulting calcium spikes may well be the adequate stimulus for the secretion of extracellular vesicles upon exercise as a basis for exercise-induced tissue cross-talk (see also Section 2.2).

The primary cilium is a unique subcellular organelle composed of nine (+0) axonema structures (Figure 2B). It carries receptor and ligand-gated channel molecules both at its membrane-associated basis, and its body, to sense fluid flow-related physical forces and translate physics into biochemistry and cell biology. Mechanical bending/deflection of the cilium was reported to enhance prostaglandin-endoperoxide synthase 2 (PTGS2 alias COX2) expression and prostaglandin production as well as bone morphogenetic protein 2 (BMP 2) and osteopontin expression. The intraflagellar transport (IFT) of components for signal transduction is apparently very relevant in this context, since the inhibition of flagellar transport 88 protein abolished this osteogenic response [22]. The role of calcium fluxes in ciliary mechanosensing is under debate. Some authors report that mechanosensing/transduction via the primary cilium is independent of calcium flux, while others state it as a plausible candidate mechanism for osteogenic-pulsed electromagnetic field stimulation [22,40]. Mechanosensing by the primary cilium was also shown to be mediated by adenylyl cyclase 6 and cyclic AMP in mice [41]. The BMP/SMAD signaling (smad proteins named after Drosophila genes, combined from “small” and “*mothers against decapentaplegic”*) as a means of mechanotransduction is also related to the cilium, since the receptor BMPIIR is located at the basal body of the cilium, and activated BMP signaling can be blocked by abolishing cilium expression. In bone, the primary cilium appears to be an important mediator of gravity and consecutive osteogenic response, because in simulated microgravity, the cilium gradually shrinks and inhibition of this shrinkage using cytochalasin D maintains osteoblast differentiation capacity (reviewed in [40]). In muscle, mechanosensing is mainly transduced via deformation strains, but still, the primary cilium has been related to satellite cell self-renewal and muscle cell differentiation in mice [42].

The downstream molecular mechanisms for signal transduction after sensing mechanical stress via the primary cilium are very much in analogy to the molecular mechanisms induced by deformation strain. On the other hand, the internal space of the cilium appears to be a separate and regulated space that sends and receives its own signals. These are mediated by the second messenger signals as well as polypeptide phosphorylation cascades, and the cilium receives and sends vesicles along a tubulin-related trafficking pathway toward the nucleus [3,9,22,43,44] (Figure 2B).

Mechanisms of mechanotransduction show vivid cross-talk with each other on their way to the nucleus and induce changes in the activation and nucleotropy of transcription factors. Consecutively transcription factor (TF)-responsive DNA elements and promoter regions get activated to modulate gene transcription that may be called “mechanoresponsive”, because the nucleotropy of respective TFs responds to strain and/or fluid flow. Such mechanoresponsive elements and promoter constructs have been analyzed and characterized by promoter bashing experiments using reporter gene constructs under the control of the iterative deletion of promoter sequences. Using cyclic stretching experiments, we investigated the mechanoresponse of the promoter of the matricellular growth factor cysteine-rich angiogenic inducer 61 (CYR61/CCN1) in human telomerase-immortalized mesenchymal stem cells [52]. Others characterized the response to the compression of a 3 kbp promoter region in the cartilage-related gene cartilage oligomeric matrix protein (COMP) [53]. Although it is known that for example, activator protein 1 (AP-1), specificity protein 1 (SP-1), and cAMP-responsive elements can rapidly be activated by mechanotransduction, much more information is required about mechanically activated DNA regions and consecutive gene regulation, both in the sense of TF activation and binding and also changes in DNA architecture and accessibility (see also Section 3.4 on epigenetics). Mechanotransduction activates early genes such as FOS, PTGS2 (alias COX2), and also CYR61/CCN1 but also moderately fast gene transcription. Transcription activation is also variably dependent on the amplitude, frequency, and duration of mechanical stimuli [52,54]. Moreover, the sensitivity to mechanical signals in bone is of course strongly modulated by other signaling molecules, as it was for example shown for estrogens and the mechano-related effects on fracture healing [15,17]. In addition, the relevance of pathways such as Hippo and YAP/TAZ signaling as well as Notch signaling in skeletal precursors and in endothelial cells has only recently been established in muscle and bone mechanotransduction [55,56,57,58]. Hence, the network of mechanosensitive signaling is getting more and more complex. The changes in transcriptomes of mechanically activated cells besides their effects on cell architecture and ECM stiffness also address their secretome, which is key for the mutual interaction of tissues at a distance via the blood circulation and body fluids (see also the following Section 2.2).

### 2.2. Systemic Mutual Interactions of Skeletal Muscle and Bone

Besides communication via physical forces/mechanosensing/adaptation mechanisms, the systemic humoral organ communication is one more classical way to interact. Secreted proteins and signaling molecules are the basis of mutual interaction between muscle and bone both in a paracrine and also an endocrine setting. This involves also other organ systems such as the central nervous system, metabolism, and putatively the whole organism. Complex interaction patterns integrate muscle and bone into such organismic interactions that still need to be unraveled in the sense of a systems biology network analysis.

#### 2.2.1. Secretion and Autocrine/Paracrine/Endocrine Communication

Secreted cell and tissue products mediate autoregulation as well as paracrine and endocrine communication between cells, tissues, and organs. A considerable part of secretory products forms the ECM and associated matricellular factors, whose adaptation is part of the mechanochemical coupling process.

Secreted products from muscle have been named myokines or exerkines (when stimulated by exercise), while products derived from bone have been called osteokines, (osteo)blastokines, or (osteo)clastokines in analogy to cytokines. Many of these products are members of huge cytokine families, but also, other growth and differentiation factors may transmit signals between muscle and bone. They are mainly related to tissue regeneration and modeling/remodeling but they also represent mediators of important metabolic functions [59,60,61,62]).

Ions and non-peptide small molecule signaling systems often supply the microenvironment to regulate intercellular communication. Prominent examples involved in tissue regeneration and mechanosensing/mechanotransduction in bone and muscle are prostaglandins, NO, sphingosines, and ATP, and its dephosphorylation-related products that fuel into purinergic signaling. Physical forces through muscular activity or vibration induce the cellular secretion of ATP and sphingosines, which are tightly involved in osteoblast, osteoclast, and osteocyte activity [2,63,64,65].

Both muscle and bone have recently been referred to as “endocrine organs” in several publications and reviews. In general, the secretion of polypeptides can follow the constitutive secretory pathway (CSP) or the regulated secretory pathway (RSP). While the former requires secretory signal peptide sequences, classical endocrine signaling requires the production of large secretory vesicles packed with preformed protein and the rapid release upon defined signals [50,51] (Figure 2C). There is no evidence that mesenchymal tissues express the tools for classical regulated secretion; the list of tissue harboring this capacity comprises truly endocrine cells of all glands, exocrine cells, neurons, immune cells, and oocytes [66,67,68,69].

Recently, extracellular vesicles (EVs) have been described as important mediators of mutual interactions both in the microenvironment and at a distance. EVs are fundamentally different from secretory vesicles that carry hormones and are involved in endocrine regulatory networks (Figure 2C). EVs can be divided into mainly two species, exosomes (50–150 nm, derived from the endosomal compartments) and microvesicles (50–1000 nm, up to 10 µm, budding from cell membranes). Their cargo ranges from miRNA over small and polypeptide molecules to organelles such as mitochondria [49]. EVs may transport myokines/osteokines, miRNA, and organelles and are involved in the regeneration of both bone and muscle tissue after exercise and injury [70,71,72,73,74]. The release of EVs upon exercise-related calcium influx may represent an important mechanism of secretion upon acute bouts of exercise and mechanotransduction. As a representative example, an in vitro study showed that osteocyte calcium oscillation as stimulated by mechanical loading induced the release of LAMP1 (lysosomal-associated membrane protein 1) containing EVs that regulate bone formation and mineralization [72]. Recently, Annibalini et al. analyzed the serum content of microvesicles in subjects ahead and 2 h after exercise and found a 2-fold increase in microvesicles of around 80–200 nm in size, indicating the putative in vivo relevance of vesicle-related information exchange between muscle and bone [74,75]. More research is needed to dissect the molecular events and to really foster the upcoming evidence.

#### 2.2.2. Bone Secretory Products with Endocrine Functions

Bone-specific secreted products can influence the formation, maintenance, and regeneration of muscle and other tissues. Many bone regulating signal transducers such as transforming growth factor β (TGFβ) and the ligands of the wnt pathway are usually thought to act more locally, but wnt3a can influence myoblast differentiation and might also act at a certain distance [76] The most prominent secreted products of bone with endocrine effects are the osteoblast marker proteins osteocalcin and the osteocyte-derived phosphatonin fibroblast growth factor 23 (FGF23) as well as the inhibitors of osteogenic wnt signaling sclerostin (SOST) and Dickkopf-related protein 1 (DKK-1).

Osteocalcin (bone gamma-carboxyglutamate protein, BGLAP) is a γ-carboxylated product of osteoblasts involved in bone mineralization and metabolic control, but also in brain development and male fertility. The degree of vitamin K-dependent γ-carboxylation of osteocalcin determines its activity in insulin sensitizing and muscular glucose uptake, where undercarboxylated or uncarboxylated osteocalcin is the most active. The injection of uncarboxylated osteocalcin in mice rescued their exercise capacity and rescued the age-associated loss of muscle mass (reviewed in [76]. G protein-coupled receptor family C group 6 member A (Gprc6a) is the receptor for osteocalcin, and its knockout in mice abolished the effect of uncarboxylated osteocalcin injections, indicating that this effect is specific for Gprc6a/osteocalcin-mediated signal transduction. Moreover, IL-6 expression was significantly downregulated in these animals, which may even more contribute to alterations in muscle regeneration.

Sclerostin (SOST) is a very potent inhibitor of osteogenic wnt signaling and appears to be rather specific to osteocytes. Recent evidence links high SOST serum levels in Korean sarcopenic women with appendicular skeletal muscle mass [77]. Exercise was reported to downregulate both SOST and DKK-1 serum levels, which marks an important link between muscle and bone via physical impact [78,79].

Glucocorticoids (GC) might be a common and potentially pathologic regulatory link between muscle and bone regeneration, since FGF-2 rescues both the deleterious effects of GC on myostatin and SOST expression [80]. Members of the FGF family exert multiple functions in bone and muscle [81,82]. The FGF23 is a member of the endocrine subfamily of FGFs that regulates phosphate excretion in the kidney and the intestinal phosphate uptake via the downregulation of 1α-hydroxylase, the vitamin D-activating gatekeeper enzyme. It is rather its indirect effect via rickets-associated myopathy that influences muscle mass and function, while its direct effects on muscle cell proliferation, differentiation, and contractility may be neglectable [83,84]. Diligent experiments by Yamamoto and colleagues have addressed the question if FGF23 is directed toward the regulated secretory pathway in osteocytes. The study showed that it bypasses this pathway, indicating that the storage granules where one can find FGF23 after osteocytic differentiation do not belong to the RSP system but are possibly just derived from the trans-Golgi-network (TGN) [85]. Hence, the putative endocrine functions of bone even for this prominent phosphatonin FGF23 rely on both constitutive secretion and vesicle release that is putatively modulated by calcium oscillations.

Hypoxia may increase the amounts of vesicles released. Various signaling compounds may also influence the respective niches of osteocytes and myocytes in response to mechanical cues, and such paracrine effects can also be transduced via vesicle fusion [32,60,61,86].

#### 2.2.3. Muscle Secretory Products with Endocrine Functions

Focusing on muscle secretory products, Bente Pedersen and her group were the first to thoroughly describe IL-6 as a putative endocrine product of muscle in response to exercise (reviewed in [87]) as well as other exercise-induced myokines [74,88]. Muscle cells, very much like bone, do not express the molecular components of the regulated secretory pathway (RSP), and the vesicle release upon exercise may be the relevant mechanism to deliver IL-6 into the circulation. Small vesicles in muscle, which contain immunoreactive IL-6 and are depleted during exercise, have indeed been described in mice [71].

The constitutive secretome of muscle (and bone cells) represents the integrated response to mechanical strain and systemic modulators. Due to less invasive accessibility, we have more information on muscle tissue compared to bone, at least in humans. Studies exploiting muscle biopsies ahead and after exercise found an enhanced transcription of characteristic target genes. The resulting changes of muscle secretomes induced by exercise occur with a decent delay to allow de novo protein synthesis, inducing a moderately fast adaptation and (re)modeling response. One example is the immediate rise of muscle insulin-like growth factor 1 (IGF-1) mRNA after an exercise bout and the significant rise of peripheral blood IGF-1 protein levels after 24 h (not yet significant after 2 h) post exercise, which was gone after 48 h [75]. IGF-1 and also FGF2 are growth factors that are actively secreted at the muscle and bone interface and if delivered into the circulation may have distant effects on bone formation and remodeling. Irisin, a cleaved product of fibronectin type III domain containing (FNDC5) with stimulating effects on bone formation, is also delivered from muscle into the circulation after exercise but may have a dual mode of delivery, shedding of preformed polypeptides and de novo synthesis [81].

Seminal work on the muscle cell secretory transcriptome and proteome was published in 2012 by Le Bihan et al. and Terry et al [89,90] Three main pathways of protein secretion by muscle cells could be identified, namely (1) conventional secretion, (2) secretion via nanovesicles with typical exosomal features, and (3) larger microvesicles (up to 200 nm in size) with distinct protein and mRNA cargo [89] (Figure 2C). The lists of muscle-related transcripts and secreted proteins comprised 149 proteins of unassigned functions as putative interactors with other tissues and 123 transcripts that corresponded to putative myokines including BMPs, chemokines, cytokines, growth/differentiation factors (GDFs), and members of the tumor necrosis factor (TNF) family of secreted proteins and ligands for Wnt signaling (Supplemental File 10 in [90]). A third report of great interest compared the immediate effects of exercise on transcription after a first bout of exercise with the effects of a similar bout after weeks of intermittent exercise where a new level of homeostasis has already been reached (Table 1). These data indicate that besides the induction of known/suspected candidate molecules, many more so far unknown candidates have to be characterized in the future [62]. A list of genes (selected by the authors using educated guesses) that are upregulated after respective time points is provided in Table 1; complete lists can be found in the article. Interestingly, the early genes such as proinflammatory cytokines IL-1b, IL-6, IL-8, the toll-like receptor 4 (TLR4) ligands serum amyloid A1 (SAA1) and SAA2, CYR61/CCN1, connective tissue growth factor (CTGF/CCN2), angiopoietin-like 4 (ANGPTL4) and ANGPTL2 very closely resemble genes that are also relevant in the initial phase of osteogenic differentiation and contribute to angiogenesis in fracture healing [91,92,93]. Already after 2h, the mRNAs encoding proinflammatory genes have gone back to basal levels. After 12 weeks of exercise, the same type of exercise bout induces a list of genes where collagens and remodeling-associated genes such as lysyl oxidase homolog 2 precursor (LOXL2), matrix-remodeling associated 5, and thrombospondin-4 (TSP-4) prevail, indicating constant ECM remodeling. TSP-4 is expressed in muscle and bone, has different functions compared to TSP-1 (antiangiogenic), and recently has been assigned to angiogenesis and matrix remodeling [94]. More in-depth functional characterization is necessary to unravel all the protein functions of putatively muscle and bone coupling genes, especially those that have so far not yet been linked to muscle and bone interaction. While this work addresses the difference between untrained versus endurance or resistance-trained healthy subjects, we have to keep in mind that the response of young versus old or healthy versus diseased exercisers may as well be fundamentally different, as it was very recently reported [95].

## 3. Common Effector Mechanisms in Muscle and Bone from Development to Postnatal Regeneration

There is evidence from genome-wide association studies that pleiotropic genes may both regulate bone and muscle homoeostasis as recently reviewed [59]. Both positive and negative correlations of loci were found in children´s genome analyses for e.g., genes active in Wnt (Wnt16, low-density lipoprotein receptor-related protein (LRP5)) and nuclear factor kappa-light-chain-enhancer of activated B cells (NFκB) signaling (TNSF11, METTL21C). Moreover, the deletion of a myocyte-related gene, mef2c, in osteocytes, causes a high bone density phenotype. These data demonstrate that there are genes whose expression is relevant for tissue formation in both bone and muscle, and others cause antagonistic effects in the brother tissues. However, this genetic evidence has to be further unraveled in terms of functional analyses.

Going back to a shared developmental origin of muscle and bone, many parallels can be found in effector mechanisms driving tissue maintenance and regeneration respectively, as discussed in the following sections.

### 3.1. Role of Common Musculoskeletal Precursor’s Mechanotransduction during Development

Muscle and bone are derived from common precursors during development, indicating a close relationship and interaction. Mesoderm (from ancient Greek μεσοσ and δερμα “between the skin”) develops from the epithelial germ layers via epithelial–mesenchymal transition (EMT), where cells disengage from tightly adherent tissue assemblies, acquire migratory capacity, and give rise to what is histologically called “mesenchyme”. This population of embryonic cells is ill-defined in terms of stemness and multipotency, but there is high plausibility that these precursors can give rise to any mesenchyme-derived tissue type including e.g., bone, cartilage, muscle, tendon, ligament, and fat tissue [98,99,100]. Moreover, some populations pass the reverse process of EMT, MET, to provide stem cells for mesengenic organ formation, e.g., adrenals, the urogenital system, and the kidneys [101,102,103]. For both EMT and MET-derived tissue types, some precursor populations develop toward “connective tissues”, possess a positional memory (based on HOX gene expression), and are important in supporting organ development. They even specify the respective organ phenotype as it is known for the intestine, pancreas, or the kidney ([102,104,105] and references therein).

When acquiring migratory capacity, mesenchyme-derived cells sense forces, respond to forces, and generate forces. The dynamic plasticity of adhesion systems and the developing cytoskeleton enables cells to migrate and provides mechanochemical interactions already at the cell–cell and cell–tissue interaction level, creating extremely complex interaction patterns [18,106].

Joint structures as important features of the musculoskeletal functional unit develop under the influence of respective core signaling cascades of osteogenic, chondrogenic, and ligamentogenic pathways. This process is fundamentally influenced by mechanical forces already in utero, directed by movement of the fetus [45,101]. In addition to mechanical signals, the fine tuning of the anatomical structure of tissues is exerted during development by a program of cellular senescence as a tool for the removal of gratuitous cells. While this ancient program plays an important role in adult life pathologies, it may also be important in adult tissue regeneration and the remodeling of tissues where it is maintained as a blueprint of developmental tissue formation [107,108]. Hence, senescence induction and cell clearance may occur without DNA damage in embryology and in regeneration and remodeling, while it shows its dark side in propagating degenerative disease when the “infection” of healthy cells and tissues occurs upon chronic cell damage and impaired clearance and drives chronic “inflammaging” (see Section 4.2) [109].

In conclusion, there is convincing evidence that during development, the interaction between physics and biology is boosted by EMT and the acquisition of migratory capacity, and every step of interaction causes and supports biological consequences in terms of lineage commitment and maturation. This early interaction via physical forces is certainly accompanied by humoral interactions; however, these are only partially unraveled in these early stages of development.

### 3.2. Common Effectors of Muscle and Bone Interaction Modulate Tissue Formation and Remodeling

#### 3.2.1. Principles of Bone Formation, Maintenance, and Regeneration

As discussed above, already very early in in skeletal development, there is evidence for an important role of mechanical forces in addition to other physical cues and soluble morphogens in controlling cellular fate determinations, patterning, and tissue development [45,46,110]. The core transcription factors orchestrating bone formation are runt-related transcription factor 2 (RUNX2) and its downstream partner osterix (OSX). Principal signaling cascades of bone formation involve canonical and non-canonical Wnt signaling, the TGFβ receptor family, and its various ligands (above all BMPs), and the parathyroid hormone receptor (PTH1R) [111].

Bone formation or modeling can be induced de novo in adult organisms by changing the impact of physical forces in everyday life such as changes of muscle forces exerted by exercise-induced hypertrophy or by changes in body weight. Modeling describes the (almost) independent activity of osteoblasts without or with only minimal osteoclast resorptive activity, just adding more mineralized tissue in response to e.g., physical forces through strain and fluid flow. The molecular cascade that follows high-impact strain to bone obviously resembles endochondral bone formation [112].

Almost any osteogenic pathway can be modulated by mechanical stimuli. Above all, the PTH1R signaling cascade is especially sensitive to mechanical loading. Anabolic PTH1R signals elicited through bone anabolic treatment using intermittent 1-34 PTH (teriparatide) are almost ineffective under conditions of unloading [113]. Besides its role in muscle physiology (see Section 3.2.2), Notch signaling has also been reported to be involved in skeletal physiology. We and others recently reported that this pathway is mechanosensitive in skeletal precursors [58,114]. Similarly, the Hippo pathway has gained attention as an important pathway in mechanotransduction and skeletal physiology. Downstream YAP/TAZ regulation is in fact tightly involved in mechanosensing and mechanotransduction both in bone and muscle as well as in many other cells, especially endothelial cells (see Section 3.3). Recently, the Ras-related GTPase RAP2 was identified to mediate mechanoresponse in this context, and it is also involved in stiffness response [55,115,116].

Bone formation and bone resorption are both part of the adaptive refinement and remodeling processes, which are mechanically guided and where both osteoblasts and osteoclasts respond to physical forces [117,118]. Coupling factors were identified e.g., in conditioned media from respective cultures added to target cells in culture. Among others, slit guidance ligand 3 (SLIT3) usherin, Fraser syndrome 1 (Fras-1), atrial natriuretic peptide, afamin, collagen α-1 chain, laminin β2, angiotensin-converting enzyme, and ADAMTS-like protein 4 were identified in a screen as osteoclast-derived compounds that stimulate osteoblast activity. Furthermore, collagen triple helix repeat containing 1 (CTHRC1) is secreted by bone-resorbing osteoclasts and stimulates osteoblast activity while at the same time suppressing adipocyte differentiation. Another important osteoclast secreted factor is sphingosine-1-phosphate (S1P), which binds to the S1PR2 receptor on osteoblasts and also stimulates the survival and migration of osteoblasts. Finally, besides SLIT3, also other axon-guided molecules such as semaphorin-4D (SEMA4D), SEMA3A, and netrin-1 were described to couple osteoblast and osteoclast activity in the remodeling process (coupling reviewed in [119]). While the role of such coupling systems in bone remodeling becomes more and more evident, systems such as sphingosine 1-phosphate and its receptor appear to be involved in mechanotransduction not only in development but also in adult life [65,120]. Bone remodeling involves the removal of gratuitous or old and damaged bone by osteoclasts ahead of tissue restoration according to (new) physical strain conditions [118]. Osteoclast activation responds to physical strain via the osteocyte receptor activator of NF-κB ligand (RANKL) production in order to restore localized microstructural heterogeneities caused by aging or new lines of forces that generate regions of “micro-unloading” [121]. In general, remodeling units can be triggered by mechanical strain and strain release, but also by cellular stress reactions and or microinjuries (microcracks in case of bone).

Next to mechanical stimuli, early bone regeneration is also associated with the secretion of proinflammatory factors showing again many parallels to the muscle regeneration mechanisms discussed below. It is supported by a proinflammatory reaction initiated by platelets in case of injury (platelet-derived growth factor (PDGF) and cytokine signaling) and/or by TLR 2 and 4 and their ligands such as SAA1 and SAA2 or cell fragments and DNA. This initiates a cascade of cytokines involving e.g., IL-1, IL-6, IL-8, and other proinflammatory polypeptides as well as Wnt5A as part of the non-canonical osteogenic Wnt pathway (see also Table 1 for their involvement in muscle regeneration) [91,122,123,124]. Not only is this condition in common for regenerating tissues in the early phase, but it also somehow mimics the senescence-associated secretory phenotype (SASP) of presenescent and senescent cells. It appears to be beneficial if intermittent, but it causes pathology if chronically active. Strategies using senolytic compounds are now issues of active research to enhance endogenous regeneration capacity [109,125,126,127].

#### 3.2.2. Principles of Muscle Formation, Regeneration, and Maintenance

During development, the formation of somites from the paraxial mesoderm is accompanied by the development of myogenic progenitor cells that give rise to all the muscles of the trunk and limbs. Somewhat analogous to the situation in bone, the head and neck muscles develop from branchial or pharyngeal arches that progressively form pharyngeal mesoderm, where many muscles (e.g., from the pharynx, jaw, and face) derive from. The orchestrating network transcription factor is paired box gene 3 (Pax3), which is the core transcription factor that initiates myogenic signaling through the family of myogenic regulatory factors (MRF), which includes MyoD, myogenin, Myf5, and MRF4. The complex myogenic differentiation process is also co-regulated by in part very specific miRNAs. Specific reviews on this process have recently been published elsewhere, but some important aspects shall be recalled in the context of muscle and bone interaction [128,129,130]. In brief, along the developmental process of embryonic primary and fetal secondary myogenesis, myoblasts become mononucleated myocytes, fuse to build multinucleated myotubes, and establish the classical myofibers just around birth and in the immediate weeks thereafter. At the time of the fetal to neonatal period, satellite cell niches are established that later on in adult life orchestrate muscle regeneration. The majority of satellite niches is established during the first 2–4 weeks of postnatal life. Satellite stem cell development in addition to Pax3 expression is largely dependent on the expression of Pax7 in the pre- and perinatal periods and modulated by mechanical strain [32]. Both Pax7-deficient and satellite cell-deficient mice fail to regenerate muscle [131].

Very much in analogy with bone and probably many other tissues, the initial phase of regeneration is governed by intrinsic inflammatory signals and is further induced by cells of the innate immune system, e.g. sequentially recruited neutrophils and resident macrophages. Cytokines already known from bone regeneration such as IL-1, 6, and 8 are involved in innate immune cell recruitment until the secretion of anti-inflammatory cytokines such as IL-10 and TGFβ1 introduce the anti-inflammatory phase of regeneration where tissue resident macrophages become important [132,133].

Notch signaling is also a crucial pathway for postnatal muscle regeneration. Muscle fibers express Notch receptors and their ligands, Delta-like ligand (Dll1, 4) and Jagged (JAG1, 2). Notch signaling in muscle follows the classical way of binding of ligand to the receptor, the intracellular cleavage of Notch by ADAM and γ-Secretase proteases, and the release of Notch intracellular domain (NICD) to the nucleus. After nuclear translocation, NICD binds the repressor RBPJ (recombination signal binding protein for immunoglobulin kappa J region), which is active in chromatin remodeling. Upon binding to NICD, RBPJ recruits chromatin remodeling complexes containing histone deacetylase or histone acetylase proteins. As a consequence of the resulting derepression, downstream Notch target genes are activated such as the transcription factors HeyL, Hes1, and Hesr1/3. This appears to be also relevant in the context of the involvement of epigenetic events where Notch signaling possibly provides links to these important upcoming fields (see Section 3.4). The relevance of the Notch signaling pathway is confirmed by the fact that the double knockout of Hes 1 and 3 causes a similar phenotype to Pax7 and RBPJ KO. However, research is still necessary to unravel the role of Notch signaling both in fate decision and morphogenesis during development and in postnatal muscle regeneration, as evidenced in dystrophic mice [131,134]. Analogous to our findings in skeletal precursors, mechanically induced Notch signaling is involved in a protein network that regulates myostatin expression in the context of cytoskeletal rearrangements via integrin-mediated AKT/PKB and mTORC1 signaling [58,135].

Satellite cells, located between the basement membrane and plasmalemma of the muscle fiber, are the principal muscle stem cells that organize muscle regeneration after injury. However, there are some other populations that substantially contribute to regeneration such as the Sca1+/CD34+ fibro-adipogenic progenitors (FAPs) residing in the muscle interstitium. Following injury, satellite cells re-enter the cell cycle and proliferate to give rise to myoblasts that differentiate and fuse to restore the damaged fiber or generate myofibers de novo, while FAPs support their amplification and fusion. Pharmacological inhibition of the latter impairs their regeneration efficiency. FAPs also harbor fibroblast differentiation capacity that in pathology may contribute to fibrosis and degeneration [131]. Recently, FAPs were reported to be involved in intramuscular fat accumulation in limb girdle muscular dystrophy and dysferlin mutation in mouse and man. Here, FAPs differentiated into adipocytes under the influence of accumulating annexin A2 (AnxA2). Since intramuscular fat accumulation can be influenced by exercise (see Section 4), these interactions in pathology may open up new perspectives for understanding disuse-related fat accumulation and its putative consequences on bone formation [136].

Overall, very much in analogy to bone regeneration, also muscle regeneration starts with an inflammatory reaction that activates the myofiber-specific amplification and differentiation process and ends up in tissue remodeling according to mechanical challenge. A condition described as fatty infiltration or degeneration in muscle through disuse and chronic inflammatory processes represents an important feature in pathology to further unravel and target also pathological interactions between both tissues and their respective mechanisms of maintenance and remodeling (see also Section 4.2).

### 3.3. Influence of Tissue Resident Cell Populations on Tissue Regeneration and Mechanoadaptation–A Focus on Immune Cell Interactions and Angiogenesis

Cells of both the innate and the adaptive immune system are intricately involved in the efficient regeneration in both muscle and bone [93,137].

Restorative macrophages even orchestrate the interaction between myogenic cells and endothelial precursors during muscle regeneration. Interestingly, the main signaling molecules involved were apelin (APLN), oncostatin M (OSM), and periostin (POSTN), which are also important for bone regeneration [138]. In addition, evidence showed that macrophage PPARƴ (peroxisome proliferator-activated receptor γ) expression controls the delivery of growth differentiation factor-3 (GDF3) and is almost essential for muscle regeneration [139]. Finally, macrophages are remarkably influenced by physical cues, including oxygen and physical forces, as to their phenotype and function, rendering them important players in the interaction of muscle and bone [140]. Recently, tissue-specific resident macrophages with specific signatures according to single cell analyses have been described to essentially support cardiac remodeling after infarction [141,142]. The transcription factor ZEB2 (zinc finger E-box-binding homeobox 2) appears to be crucial for the tissue-specific dissemination of macrophages, while in the liver, the coexpression of ZEB2 and LXRα (liver X receptor α) is required to maintain the local specific Kupffer cell population. This finding may raise exciting perspectives for other tissues such as muscle and bone. In the latter, so far, mainly osteoclasts have attracted attention as the most important candidates of the monocyte/macrophage lineage, but recent evidence also suggests the importance of resident macrophages while it is not completely clear if they originally are derived from liver or from bone marrow [143,144,145].

Endothelial cells (EC) are essential for tissue regeneration. For bone, it has been demonstrated that endothelial specification strongly contributes to microarchitectural structures—for example, cortical versus trabecular bone. Moreover, there is again an important contribution of Notch signaling since the process of endothelium specification and its mutual interaction with osteogenesis is Notch dependent [146,147,148,149,150]. Almost identical associations have been reported for the muscle regeneration process. Both populations secrete proangiogenic molecules such as APLN, OSM, and POSTN. They influence motility and the tube formation process in vitro and in vivo, indicating that both the effects of innate immune cells and EC do mutually interact in bone and muscle regeneration and use a remarkably overlapping panel of signaling pathways. Even more, in what may be a kind of standby mechanism in case regeneration is required, muscle satellite cells attract and cross-talk with EC via vascular endothelial growth factor (VEGF) secretion, while the EC-derived Notch ligand Dll4 causes muscle stem cell quiescence and creates a vascular niche [151]. EC do strongly respond to mechanical cues, and one important target for their mechanoresponse is again the Notch pathway [152]. Although ectodomain shedding of jagged and Notch is a well-known phenomenon, there are no reports about the humoral modulation of this local cell–cell interaction regulatory system, limiting—in the case of Notch signaling—the interaction between muscle and bone to mere mechanically induced mechanisms.

Overall, tissue regeneration appears to be a teamwork that requires the interactions of several cell populations including tissue-specific precursors, immune cells, and endothelial precursors, whereby also the latter seem to be tissue specific, and this applies to both muscle and bone [153].

### 3.4. Epigenetic Changes Shape Individual Responses According to Lifestyle, Environment, and Aging

Epigenetic changes are part of the physiological tissue responses on mechanical stimulation and pathology, but they may also fundamentally change and often impair the response patterns. The general feature of epigenetic regulation, the regulation of DNA domain accessibility for transcription factors (TFs), is shaped by high-order chromatin interactions. However, some recent observations indicate that some TFs actively contribute to the modeling of DNA architecture, as it was reported for MyoD. This TF can obviously change nuclear architecture to dominantly set up the muscle cell differentiation program even in a preformed environment through cooperation with the architectural protein CTCF (CCCTC-binding factor) [154]. Intriguingly, the vitamin D receptor (VDR), an important steroid hormone receptor in both muscle and bone, also fundamentally changes DNA architecture via CTCF interaction [155,156]. Moreover, the local delivery of VDR ligands via vitamin D metabolizing enzymes is influenced by power exercise in a rat model, providing very strong links between muscle and bone interaction and epigenetics [157]. Such a physiologically relevant modulation of regeneration can be disrupted by aging phenomena, which largely rely on epigenetic changes that change the signatures in regenerative cells. As an important example, a recent publication reports that α-Klotho expression in regenerating muscle after injury is intermittently enhanced by promoter demethylation. This process is blunted in aged mice, indicating a relevant modulation of muscle regeneration through the epigenetically driven alteration of muscle regenerative capacity [158]. In case of bone, we and others have recently reported that there are no significant associations to epigenetic marks or aging associated DNA signatures in peripheral blood mononuclear cells in age-related osteoporosis, while others claim to see such associations, and the debate is ongoing [159,160,161]. What appears to be quite obvious is that skeletal precursors obtained from the bone marrow of osteoporotic patients harbor an almost diagnostic signature of their transcriptome, while the putatively epigenetic hubs of regulation await further identification [162]. Such tissue-specific segmental epigenetic changes may play important roles in disease, and we definitely need more research in this area. With respect to exercise as a countermeasure of bone and muscle loss, there is evidence that exercise is followed by epigenetic changes that besides modulating the accessibility of chromatin regions for adaptive signaling even determine the availability of metabolic intermediates for acetylation and methylation. Moreover, many of these epigenetic effects remain as signatures that may even have a transgenerational impact [163,164].

### 3.5. Conclusions–Two Systems of Mutual Communication between Muscle and Bone

In conclusion, there are basically two systems to mutually communicate between muscle and bone where physics and biochemistry meet. Physical forces exerted by physical load, muscle activity (contraction and relaxation), and fluid flow are translated into biochemical signals and the biology of cells and tissues. The primary biological response to physical cues changes the receiving biological system, and in consequence, the secondary response is different until homeostasis is achieved, while decreasing physical challenge causes tissue degradation in both compartments. This is the basis for a continuum of reciprocity that is even more complex, since cells and organisms can generate resistive and propulsive forces, especially in the context of migration and locomotion. A highly complex but also very economic system of adaptation models the living organism according to environmental changes. Having said this, it becomes evident that the same regulatory mechanisms are very sensitive to malfunction and the development of disease if the conditions of life are rapidly escaping millions of years of evolution and are rapidly changing such as “unnecessarily” long life spans or an exuberance of nutrients without the necessity of moving for their retrieval. It is not possible to provide an in-depth review of any pathology in the context of muscle and bone interaction, but the following two sections will look at two aspects of interaction that appear to be important in the context of development of osteoporosis and sarcopenia, namely chronic inflammation and unloading.

## 4. Muscle and Bone Interactions in Disease—Lessons and Backflashes from Pathology

Muscle and bone in concerted action with joint and ligament structures represent the musculoskeletal functional unit. Many disease entities point toward a strong interaction of the formation, maintenance, and remodeling of all components through the interaction of individual pathologies. Single component diseases are widespread, as it is the case for osteoporosis, sarcopenia, osteoarthritis, and degenerative tendon disease. It is beyond the scope of this review to describe all aspects of interaction. When focusing on muscle and bone, the most obvious mechanisms that trigger diseases in both compartments are unloading and chronic inflammation, which will be discussed in the following sections for translational perspectives.

### 4.1. Unloading–Lessons from Bed Rest Studies and Microgravity

Disuse in the sense of low mobility-related or mechanical unloading is discussed as one of the most predominant problems in modern societies, although in young people, such effects are not (yet) found to be very prominent [12,165]. Yet, the unloading of muscle and skeleton causes muscle wasting and bone loss even in younger subjects. Since this condition is extremely relevant in space missions, a lot of research has been conducted in this context using e.g., simulated microgravity (µG, bed-rest studies), which taught us a lot of the effects and the mechanisms of unloading versus loading in the physiological interactions of muscle and bone with a focus on mechanical forces. A recently published meta-analysis including 75 µG simulation studies reported moderate changes on muscle parameters within 7–14 days of µG and large effects on muscle force, mass, and peak power after 35 days [166]. A series of carefully designed studies on the effects of serum biomarkers for muscle and bone turnover regulation has been conducted, which confirmed that a continuous loss of both muscle and bone mass could be observed during simulated µG. Long-term bed-rest µG simulation over 90 days 6° head-down tilt was followed by a decrease of a muscle fiber cross-sectional area of 26% [167]. This process was accompanied by an increase of collagen resorption marker peptides and an increase in the powerful muscle and bone formation inhibitors SOST and myostatin, which was reversible upon reloading [168,169]. Careful studies on the molecular mechanisms revealed that the majority of regulated genes and proteins were related to insulin resistance, metabolism, and reduced protein synthesis [170,171,172]. As expected, the adenosine monophosphate-activated protein kinase (AMPK) that functions as a main signal transduction mechanism for muscle hypertrophy was identified [173]. A putative candidate for the interactive effects on muscle and bone is IGF-1 and the downstream cascade of PI3 kinase/AKT signaling (phosphoinositide 3-kinase/ serine/threonine kinase signaling), which is also regulated by disuse versus exercise [174,175]. Mechanosensitive ion channels, which were discussed above as important generators of calcium spikes for osteogenic signals, are altered in µG as shown for neuronal cells or the cells of primitive organisms. Although there are not many data about musculoskeletal systems, one should anticipate that the same holds true for musculoskeletal signal transduction [176]. Many of the genes in the respectively published gene lists remain to be characterized in terms of their functional relevance for mechanotransduction and the maintenance of muscle and bone mass and function. Spaceflight observations are proof of principle test conditions for Zero-G, and such reports confirm the rapid loss of muscle volume and all types of muscle fibers even in short-term spaceflights already after a short time in space of 5–111 days (reviewed in [170].

### 4.2. Inflammation and Fatty Degeneration—Lessons from Imaging and Structural Analyses

Chronic inflammation can be both a cause and consequence of disuse and atrophy in muscle and bone. It impairs mechanotransduction and triggers the production of inhibitors of regeneration in both compartments, namely myostatin, activin, SOST, and DKK-1 under various clinical conditions. Apart from chronic inflammatory rheumatic and intestinal diseases, so-called subclinical inflammation is also a driver of tissue-related “inflammaging”. This form of inflammation can seriously impair tissue regeneration on several levels and in several segments. While inflammation is an essential part of the initial phase of tissue regeneration, the failure of timely resolution through anti-inflammatory cytokines is key for these roadblocks and it also drives aging and the manifestations of an inflammatory senescence-associated phenotype (SASP) [91,107,108,109,177]. Inflammation and disuse also propagate muscle fatty infiltration and bone marrow adipose expansion, which even more contributes to inflammatory activity initiating a vicious cycle. Exercise and mechanotransduction can be a countermeasure for this type of chronic inflammatory tissue degeneration. New imaging techniques can demonstrate this interaction by quantitating intramuscular adipose tissue before and after exercise intervention and when combined with structural analyses may also impressively demonstrate the interactions between muscle and bone in pathology.

Dual x-ray absorptiometry (DXA) and bioimpedance analysis (BIA) are widely and easy-to-use techniques to quantify body composition [178]. The former provides a quantitative measurement of lean (but not of muscle) mass and of fat mass of the total body or a sub part of it (Table 2). The initial definition of sarcopenia by Baumgartner was based on appendicular lean as measured by DXA [179]. Bioimpedance analysis systems estimate fat and fat-free mass from the electrical impedance measurements of trunk, arms, and legs using age, gender, and ethnic-specific algorithms validated in previous studies, albeit with large prediction errors for individuals [180]. Neither DXA nor BIA can provide three-dimensional (3D) measurements. This is the domain of computed tomography (CT) and magnetic resonance imaging (MRI).

**Figure 3:**
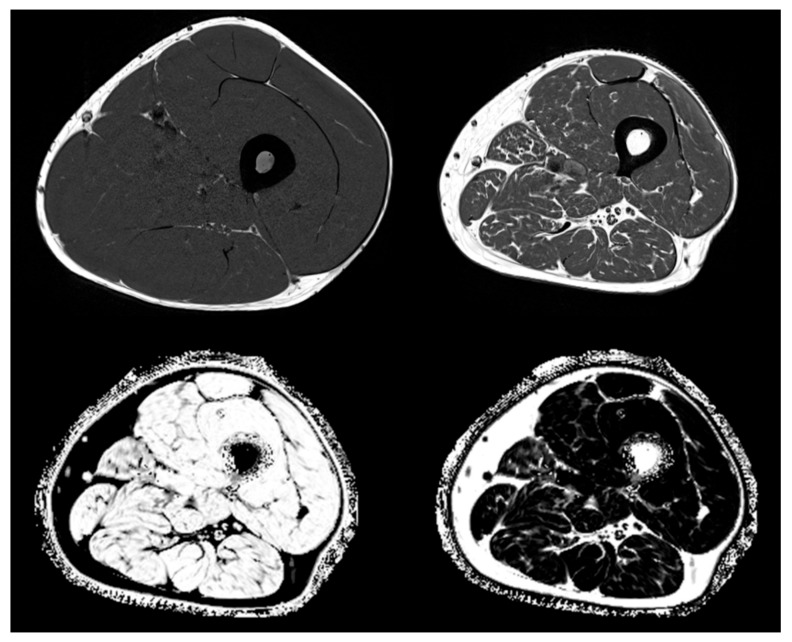
Magnetic resonance imaging (MRI)-based muscle imaging. Weighted image of young healthy (top left) and elderly sarcopenic male (top right). Water fraction (bottom left) and fat fraction (bottom right) images of subject shown in top right.

**Figure 4:**
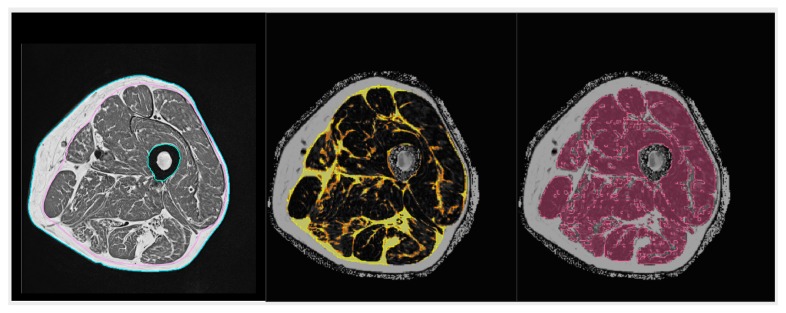
Segmentation of MRI (magnetic resonance imaging) images. left: T1 image used for the segmentation of the fascia lata (red) separating the subcutaneous adipose tissue from the group of muscles and IMAT; center: Dixon fat fraction (FF) image with segmented: muscles (black) and IMAT (yellow and red), smaller agglomerations of extramyocellular lipids (EMCL) are shown in red; right: remaining muscle tissue for which FF is determined (Images from University of Erlangen).

In clinical routine, the preferred method is semi-quantitative assessment of muscle and adipose tissue area or volume from T1 weighted MR images, which show very good contrast between these two tissues (see Figure 3 top left and top right showing T1 weighted images from a young healthy normal male and from an elderly male with sarcopenia). T1 images are used for the assessment of intermuscular adipose tissue (IMAT), which is the ensemble of ‘white voxels’ within the fascia lata (FL) [181]. White voxels in the T1 images show adipocyte agglomerations located between as well as within individual muscles. Within the muscles, they are located among individual muscle fibers and are often summarized as extramyocellular lipids (EMCL) in contrast to intramyocellular lipids (IMCL), which are droplets of triglycerides located within muscle cells. In essence, IMAT denotes larger agglomerations of EMCL and perimuscular adipocytes within the FL. Intra- and extramyocellular lipids, IMCL and smaller EMCL agglomerations, are not captured by IMAT as measured by MRI (Figure 4).

With MR Dixon sequences that generate so-called fat and water fraction images (Figure 3 bottom) [182], the fat fraction which is the combined %fat of the analyzed VOI and can be quantified. For example, in muscle, FF reflects all EMCL and IMCL. Differentiation between these components is only possible with MR spectroscopy (MRS) [183,184], which is not an imaging method.

Computed tomography is an alternative 3D modality for muscle imaging [185]. Similar to MRI T1 weighted imaging, it can be used to measure muscle and IMAT area or volume. In analogy to Dixon imaging, with CT, a muscle density measured in Hounsfield units (HU) (0 HU for water and–1000 HU for air) can be quantified. The value of muscle tissue (about 50–60 HU) is lowered by the infiltration of lipids. Interestingly, to the best of our knowledge, a direct comparison of CT muscle density with MRI Dixon FF results has not been published so far.

Table 2 presents an overview of the muscle and fat parameters obtained with the different modalities introduced above. None of the modalities can actually measure muscle mass, but lean mass and muscle volume are used as surrogates. The total body and abdominal measurements are the basis of body composition assessments, which, often combined with nutritional aspects, are integrated into clinical routine and have been used for the diagnosis and monitoring of disease progression in oncology, diabetes, obesity, sarcopenia, and cardiovascular [186,187,188,189]. However, there is a disassociation between muscle strength or power and lean mass or muscle size with respect to exercise intervention and age-dependent changes, because the contractile muscle capacity is strongly influenced by the EMCL and IMCL distribution [190,191,192,193,194]. In consequence, a quantitative assessment of the muscle composition has been suggested [195]. Here, the preferred imaging location is the thigh. The quantification of the muscle composition requires sophisticated image processing to separate subcutaneous adipose tissue (SAT) from IMAT and muscles.

Currently, there are no guidelines by which imaging parameter(s) should be quantified to address a given muscle composition question. For example, whether IMAT or muscle FF are more sensitive, parameters or whether the FF of all or of specific muscles should be measured. In the context of osteoporosis muscles, in addition to the thigh and hip region, paraspinal muscles are of great interest in order to describe the muscular compartments around bones affected by so-called major osteoporotic fractures. Comparative studies are still missing.

The concept of bone muscle interaction should have an impact on osteoporotic fracture. In men, a decrease in subcutaneous fat thickness increased hip fracture risk independent of area bone mineral density (BMD) [196], but in women, independent contributions of DXA fat or lean measurements on fracture risk could not be shown so far [197,198,199]. More positive results have been reported from CT studies analyzing muscle attenuation and size [200,201]. In a more recent retrospective analysis of the European Femur Fracture Study (EFFECT), a cross-sectional study of 40 female patients with acute hip fractures and 55 controls, we performed a sophisticated texture analysis of the distribution of EMCL and muscle tissue [202] using a CT density-based histogram analysis was performed [203]. After adjustment for BMD and cortical thickness, the hip fracture group was characterized by the lower relative adipose tissue volume of the upper thigh and some of the texture parameters discriminated the osteoporotic hip fracture [204]. The results could be interpreted as representing bone strength, protection against hip fracture by a larger cushion, and muscle degeneration.

Interestingly, the combination of soft tissue parameters discriminated hip fractures as well as a combination of trabecular BMD and cortical thickness, documenting a strong interaction between muscle and bone structure using fatty infiltration and other soft tissue architectural parameters as surrogate parameters. The multimodality imaging of CT and MR or the combination of different MR sequences including the integration of T2 imaging for the quantification of inflammation along with the development of sophisticated image processing and analysis techniques will further advance our understanding of muscle composition as surrogates for actual power and fatty infiltration-based inflammatory activity and their impact on bone density, architecture, and fracture resistance.

## 5. Conclusions and Perspectives

Muscle and bone interact in order to provide adaptive modeling and remodeling mechanisms in an environment that is physically challenging. Both tissues sense mechanical strain, transduce biochemical signals in response, and mutually influence the respective tissue architecture to balance muscle power and fracture resistance. Ongoing intense research activity dissects the molecular mechanisms that transform physics into biochemistry and cell biology. It is physical strain that strongly modulates the biology of cells along their lineage commitment and maturation as well as the hypertrophy versus atrophy of tissues according to physical challenge. Physical strain is transformed into a multitude of biochemical signals that causes modeling or remodeling until a new state of homeostasis is reached as long as the physical input remains in a steady state. Decreasing physical input leads to muscle atrophy and bone loss, as is most impressively documented under zero-G in space missions. We are beginning to understand the mechanisms of regulation, but we do not yet exactly know where the borders to pathology begin, which stimulus hurts cells, and which one stimulates adaptive remodeling. We need more research in this area to define the borders between benefit and harm both in everyday life and in sports activities. Backflashes from unusual unloading situations as well as from pathologic inhibitory conditions such as those in chronic inflammation indicate that deficient mechanotransduction is both a driver and a consequence of degenerative diseases such as osteoporosis, sarcopenia, and chronic degenerative tendon conditions. Continuous adaptive remodeling appears to be the key to delay aging-associated degeneration; hence, exercise and locomotion in concerted interaction between muscle and bone are core phenomena of health maintenance in higher organisms. Epigenetics is strongly involved in aging phenomena, and recent research indicates that both positive exercise and training effects and also exercise resistance and degeneration can be modulated by epigenetic mechanisms. As we get older, such conditions prevail and the dissection of the molecular mechanisms behind will allow for more targeted prevention and intervention in the future.

## Figures and Tables

**Table 1:** Exercise-related upregulation of genes in muscle biopsies after 45 min exercise, 2 h after 45 min exercise, and 12 weeks after intermittent exercise according to Pourteymour et al. [62]. Many of these genes have putative functions in bone regeneration (see also text). Virtually all genes of the inflammatory phase have implications in the early phase of skeletal stem cell commitment and bone regeneration; also, genes active in neoangiogenesis are relevant for bone healing, regeneration, and remodeling [82,91,96,97]. Abbreviations: CYR61/CCN1: cysteine-rich angiogenic inducer 61, TNF: tumor necrosis factor.

Inflammatory Phase Upregulated Genes after 45′ Exercise	Upregulated Genes 2 h after 45′ Exercise	Upregulated Genes after 12 Weeks of Intermittent Exercise
Pourteymour et al. [62]		
Interleukin 6	Interleukin 6 receptor	Secreted frizzled-related protein 5
Interleukin 8	Colony stimulating factor 3 receptor	Secreted frizzled-related protein 2
Interleukin 1, beta	TNF receptor superfamily member 8	
Prostaglandin–endoperoxide synthase 2	Prostaglandin I2 (prostacyclin) receptor	
Chemokine (C-X-C motif) ligand 1	Tumor necrosis factor receptor	Chemokine (C-C motif) ligand 21
Chemokine (C-C motif) ligand 8	Complement component 8	Collagen, type I, alpha 1
Chemokine (C-X-C motif) ligand 2	Plasminogen	Collagen, type III, alpha 1
Chemokine (C-C motif) ligand 2	Stanniocalcin 2	Collagen, type IV, alpha 1
Chemokine (C-X-C motif) ligand 3	Lipocalin 10	Collagen, type IV, alpha 2
Chemokine (C-X3-C motif) ligand 1	Lipocalin 6	Collagen, type VI, alpha 6
Leukemia inhibitory factor		Lysyl oxidase-like 2
Serum amyloid A1		Matrix-remodeling associated 5
Serum amyloid A2		Osteoglycin
Angiopoietin-like 4	Angiopoietin-like 4	Biglycan
CYR61/CCN1	Angiopoietin-like 2	
Connective tissue growth factor/CCN2		
Vascular endothelial growth factor A		
Thrombospondin 1	Thrombospondin 1	Thrombospondin 4
Fibroblast growth factor 6	Fibroblast growth factor 6	Insulin-like growth factor 2
Fibroblast growth factor 18		
Matrix metallopeptidase 19	Serpin peptidase inhibitor, clade F, member 2	
ADAM metallopeptidase with thrombospondin type 1 motif 4	Serpin peptidase inhibitor, clade A, member 3	
ADAM metallopeptidase with thrombospondin type 1 motif, 1	Serpin peptidase inhibitor, clade A, member 1	
	ADAM metallopeptidase with thrombospondin type 1 motif 9	

**Table 2:** Standard anatomical locations (VOI: volume of interest) and standard parameters quantified by dual x-ray absorptiometry (DXA), bioelectrical impedance (BIA), magnetic resonance imaging (MRI) using T1-based or Dixon sequences and computed tomography (CT) in muscle and body composition assessments. HU: Hounsfield units; SAT: subcutaneous adipose tissue; VAT: visceral adipose tissue; IMAT: intermuscular adipose tissue; FF: fat fraction; WF: water fraction. HU: Hounsfield Units.

	VOIs	Parameters
DXA	total body appendicular skeleton abdomen: estimation of SAT and VAT	lean mass (g) fat mass (g)
BIA	total body appendicular skeleton abdomen: estimation of SAT and VAT	estimates of lean and fat mass (g) based on sex, age, and ethnicity-specific equations
MRI T1	thigh abdomen paraspinal muscle	SAT, VAT area/volume (cm^2^/cm^3^) IMAT area/volume (cm^2^/cm^3^) muscle area/volume (cm^2^/cm^3^)
MRI Dixon	whole body thigh paraspinal muscle	FF (%) WF (%)
CT	thigh abdomen paraspinal muscle	SAT, VAT area/volume (cm^2^/cm^3^) IMAT area/volume (cm^2^/cm^3^) muscle area/volume (cm^2^/cm^3^) muscle density (HU)
